# Enhancing Cookie Formulations with Combined Dehydrated Peach: A Machine Learning Approach for Technological Quality Assessment and Optimization

**DOI:** 10.3390/foods13050782

**Published:** 2024-03-02

**Authors:** Biljana Lončar, Lato Pezo, Violeta Knežević, Milica Nićetin, Jelena Filipović, Marko Petković, Vladimir Filipović

**Affiliations:** 1 Faculty of Technology Novi Sad, University of Novi Sad, Bulevar Cara Lazara 1, 21000 Novi Sad, Serbia; ovioleta@uns.ac.rs (V.K.); milican@uns.ac.rs (M.N.); vladaf@uns.ac.rs (V.F.); 2 Institute of General and Physical Chemistry, Studentski trg 12/V, 11000 Belgrade, Serbia; latopezo@yahoo.co.uk; 3 Institute of Food Technology in Novi Sad, University of Novi Sad, Bulevar Cara Lazara 1, 21000 Novi Sad, Serbia; jelena.filipovic@fins.uns.ac.rs; 4 Faculty of Agronomy, University of Kragujevac, Cara Dušana 34, 32102 Čačak, Serbia; marko.petkovic@kg.ac.rs

**Keywords:** support vector machine, Artificial Neural Network, modeling data, peach addition, cookie quality

## Abstract

This study focuses on predicting and optimizing the quality parameters of cookies enriched with dehydrated peach through the application of Support Vector Machine (SVM) and Artificial Neural Network (ANN) models. The purpose of the study is to employ advanced machine learning techniques to understand the intricate relationships between input parameters, such as the presence of dehydrated peach and treatment methods (lyophilization and lyophilization with osmotic pretreatment), and output variables representing various quality aspects of cookies. For each of the 32 outputs, including the parameters of the basic chemical compositions of the cookie samples, selected mineral contents, moisture contents, baking characteristics, color properties, sensorial attributes, and antioxidant properties, separate models were constructed using SVMs and ANNs. Results showcase the efficiency of ANN models in predicting a diverse set of quality parameters with r^2^ up to 1.000, with SVM models exhibiting slightly higher coefficients of determination for specific variables with r^2^ reaching 0.981. The sensitivity analysis underscores the pivotal role of dehydrated peach and the positive influence of osmotic pretreatment on specific compositional attributes. Utilizing established Artificial Neural Network models, multi-objective optimization was conducted, revealing optimal formulation and factor values in cookie quality optimization. The optimal quantity of lyophilized peach with osmotic pretreatment for the cookie formulation was identified as 15%.

## 1. Introduction

Cookies are top-rated baked products that are highly accepted by consumers, despite being primarily made of butter, refined sugar, and wheat flour, which makes them high in calories and low in dietary fiber and bioactive compounds from a nutritional standpoint [1,2].

Currently, a significant number of consumers are cognizant of the connection between insufficient nutrition and the onset of diet-related disorders, leading them to actively seek functional foods that are nutrient-rich and have the potential to positively impact their physiological well-being [3,4]. Incorporating fruit or fruit by-products into cookie formulations led to increases in the fiber contents and bioactive compounds of the cookies [5]. In determining the precise level of each novel ingredient and changing the standard cookie dough, it is essential to consider health aspects, technological quality, and consumer acceptability, especially as the inclusion of new additives in cookie formulations requires meticulous testing and quality optimization [5,6].

Integrating machine learning models plays a crucial role in modeling and optimizing formulations in food applications [7]. These advanced computational tools, including Support Vector Machines (SVMs) and Artificial Neural Networks (ANNs), contribute to a more precise understanding of ingredient interactions and their impact on product attributes [8]. By leveraging machine learning algorithms, researchers can model complex relationships within diverse data sets and optimize food formulations [9,10].

Utilizing SVMs for regression analysis involves employing hyperplane classifiers that map input data into a multidimensional space, enabling comparison with the output data [11,12]. The research conducted by Nirere et al. [13] showed the feasibility of employing hyperspectral imaging technology in conjunction with the least-squares support vector machine model for classifying the quality of dried wolfberry fruit.

Recently, ANNs have been increasingly used in modeling food product formulations, particularly through the efficiency of multilayer perceptron (MLP-ANN) in regression applications [14,15]. ANNs, characterized by their structure, learning algorithm, and activation function, effectively fit and generalize data based on selected input parameters to achieve desired output values [16]. The effectiveness of ANNs is assessed by comparing experimental and computed data [17].

Adaptable non-linear methods serve as powerful tools for modeling intricate relationships in diverse datasets from instrumental analysis and are extensively employed in food analysis for classification, optimization, and regression investigations; numerous examples demonstrate their ability to achieve high-quality results that surpass those available from traditional methods in some cases [18,19].

**Figure 1:**
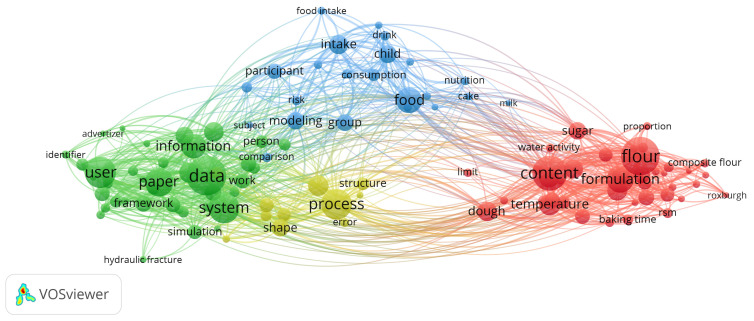
Co-occurrence analysis of cookie optimization and modeling metadata of abstracts from Scopus.

The VOSviewer program was employed to detect patterns in scientific papers related to cookie optimization and modeling, utilizing author and index keywords for representation. A comprehensive analysis was carried out by searching through abstracts 350 times. The co-occurrence analysis of metadata on cookie optimization and modeling revealed four distinct groups, as illustrated in Figure 1.

Cookie formulation and dough preparation-related words were collected in the red cluster, with the words “flour”, “formulation”, “dough” and “sugar” being the most frequently mentioned in the summaries analyzed. The green cluster included data system and information parameters applied in cookie optimization and modeling. The most frequent terms in the green cluster were “data”, “user”, “information”, “paper”, and “simulation”. The yellow cluster summarized processes of cookie production, optimization, and modeling, and the most frequently used terms were “process”, “shape”, “structure”, and “error”. The blue group included terms regarding food modeling. The most frequently used terms were “food”, “modeling”, “consumption”, “intake”, and “group”.

The frequency of occurrence of each word is depicted by the size of the circle. Diverse colors were used to delineate distinct clusters of closely interconnected keywords, facilitating their classification. VOSviewer software ver. 1.6.20 was employed to elucidate the phrase structure, utilizing data obtained from the Scopus database [20]. Presently, there is often a deficiency in comprehensive examinations of the intricate relationships within the realm of cookie optimization and modeling in current research.

There is a need for thorough exploration of the impacts that cookie optimization and modeling parameters have on the quality of end products. Additionally, the refinement of cookie optimization and modeling through the application of mathematical models remains an area deserving of more extensive investigation. Addressing these gaps in knowledge is necessary for advancements in this field and for fully harnessing the capabilities of mathematical modeling in enhancing the efficiency of cookie optimization and modeling processes.

In this study, nonlinear machine learning models, specifically SVMs and ANNs, were constructed. The input parameters for these models were the percentage of dehydrated peach addition and the method of preparation of the dehydrated peach, which included both the lyophilization method and lyophilization with osmotic pretreatment in sugar beet molasses. The data for the modeling were taken from our previously published paper [21] as a continuation of the research.

The primary objective was to identify the most suitable machine learning model, not only in terms of predictive accuracy but also considering the error rate of each model. Comparison of the models involved assessing different modeling errors by juxtaposing literature findings and calculated data with the model outcomes [22]. The main parameter for model comparison was the coefficient of determination, which serves as the key metric to identify the most suitable model for estimating overall technological quality [23].

Yoon’s global sensitivity method, utilizing the ANN model, was employed to explore the impact of input variables (percentage of peach addition and treatment used) on output variables (cookies’ chemical, mineral matter, and phenolic compound contents, the antioxidative activity of nutritive parameters, and the physical, technological, textural, color, and sensory characteristics of the technological parameters).

The overarching aim was to predict and optimize the percentage of peach addition to cookie formulations, focusing on the overall technological quality aspect.

## 2. Materials and Methods

### 2.1. Experimental Data

As a logical extension of prior investigations, we utilized experimental data from our previous study [21]. In brief, cookie samples featuring varying levels of dehydrated peach addition were tested for their chemical, mineral matter, and phenolic compound contents, the antioxidative activity of nutritive parameters, and the physical, technological, textural, color, and sensory characteristics of the technological quality. Peaches underwent lyophilization and lyophilization with osmotic pretreatment in sugar beet molasses solution for 5 h at 20 °C. Subsequently, the dehydrated peach samples were incorporated into cookie formulations, replacing flour at levels ranging from 0% to 25%.

**Figure 2:**
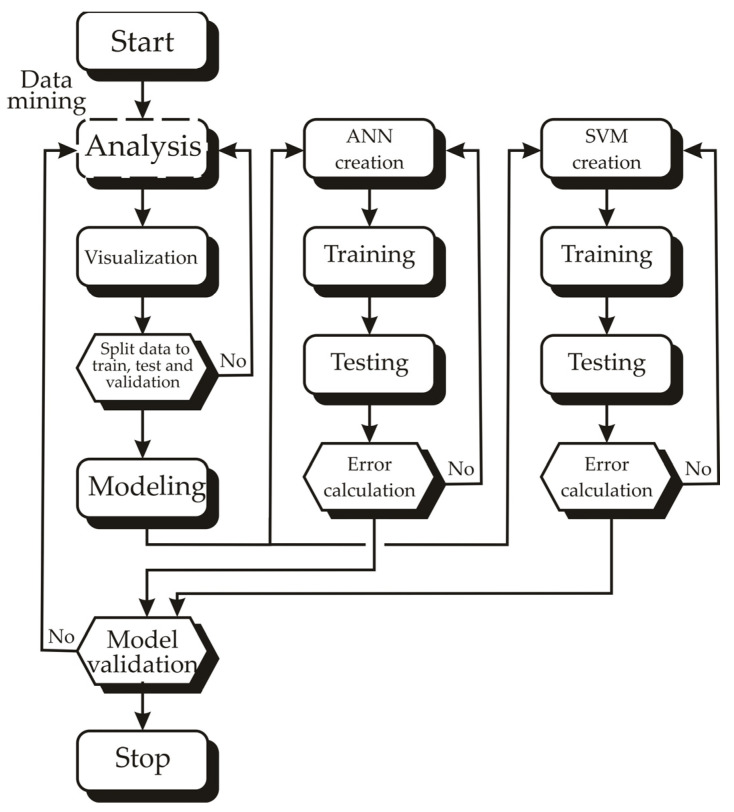
Flowchart of the conducted research.

To enhance comprehension of the research framework, Figure 2 illustrates the flowchart detailing the conducted investigation. 

### 2.2. SVM Modeling

Support Vector Machine (SVM) models rely on averaging principles and serve as algorithms applicable to supervised learning in regression tasks. In the context of regression, SVM models predict outcomes by partitioning the data into segments for model training and testing. These models are well-suited for forecasting various output variables, including the chemical compositions of cookies (such as mineral matter and phenolic compounds), the antioxidative activity of nutritional parameters, and the physical, technological, textural, color, and sensory characteristics of the technological parameters. 

In nonlinear applications, the initial step involves transforming input vectors from a low-dimensional space using a nonlinear function in the modeling process (1) (*Φ*) [24]:

(1) $$\left(x\right)=w^{T}\cdot \Phi \left(x\right)+b$$

where *w* and *b* represent the weight vector and intercept of the model.

The SVM models were developed to predict output variables based on the input parameters (percentage of peach addition and treatment used). The model was created as regression type 1 with a training constant of 10. The epsilon measure of the model was set to 0.1, while the radial basis function (gamma value) was set to 1.00. The total number of model iterations was 10,000.

### 2.3. ANN Modeling

Artificial Neural Network (ANN) modeling, specifically Multi-Layer Perceptron (MLP), is a prominent type of ANN that is extensively employed in computing [25]. The key strength of this network lies in its ability to “learn” and establish connections between input and output data [26,27]. This feature proves highly valuable for predicting nonlinear problems, especially in domains requiring the processing of substantial volumes of data [28]. The quantity of artificial neurons within the hidden layer is subject to variation depending on the error and trial methods employed. In the learning process of a neural network, input data undergo processing, ultimately leading to the conversion of the input data into the desired output data [29]. There are two fundamental types of network learning processes: supervised and unsupervised. In supervised learning, the model is supplied with Artificial Neural Network (ANN) output data, allowing it to compare and assess the values obtained during the learning process [30]. The data basis was divided into 70% training and 30% testing data. The developed ANN models were trained 100,000 times per model with a random number of neurons in the hidden layer (3–15). Various activation functions and randomly assigned values for weighting coefficients and biases were employed in the modeling process. To address nonlinear optimization challenges, the Broyden–Fletcher–Goldfarb–Shanno (BFGS) algorithm was utilized. This algorithm played a key role in optimizing the parameters of the Artificial Neural Network (ANN) during the modeling process [31]. The neural network models, expressed in matrix notation, incorporate biases and weight coefficients for the hidden and output layers. These are symbolized by the matrices and vectors *W*_1_, *B*_1_, *W*_2_, and *B*_2_, where *W*_1_ and *W*_2_ are matrices for the weight coefficients, and *B*_1_ and *B*_2_ are vectors representing biases. The output value, denoted as *Y*, is determined by transfer functions, with *f*_1_ representing the transfer function for the hidden layer and *f*_2_ for the output layer. The input layer matrix is denoted by *X* [32,33]:

(2) $$Y=f_{1}\left(W_{2}\cdot f_{2}\left(W_{1}\cdot X\right)+B_{1}\right)+B_{2}$$

### 2.4. Global Sensitivity Analysis

Yoon’s interpretation method was employed to assess and quantify the relative impact or influence of the input variables (percentage of peach addition and treatment used) on output variables (cookies’ chemical, mineral matter, and phenolic compound contents, the antioxidative activity of nutritive parameters, and the physical, technological, textural, color, and sensory characteristics of the technological parameters). These calculations were performed according to the weight coefficients of the developed ANN models [34]. The given equation was utilized to evaluate the direct influence of the input parameters on the output variables, taking into account the weighting coefficients incorporated within the Artificial Neural Network (ANN) models [35]:

(3) $$RI_{ij}\left(\%\right)=\frac{\sum_{k=0}^{n}\left(w_{ik}\cdot w_{kj}\right)}{\sum_{i=0}^{m}\left|\sum_{k=0}^{n}\left(w_{ik}\cdot w_{kj}\right)\right|}\cdot 100\%$$

where *w* represents the weights of the ANN model, *i* is the input variable, *j* is the output variable, *k* is the hidden neuron, *n* is the number of hidden neurons, and *m* is the number of inputs.

### 2.5. The Accuracy of the Models

To gauge the efficacy and performance of the Support Vector Machine (SVM) and Artificial Neural Network (ANN) models in predicting output variables from input data, various statistical parameters were computed. These parameters encompassed the reduced chi-square (Χ^2^) (4), root mean square error (RMSE) (5), mean systematic error (MBE) (6), mean percentage error (MPE) (7), total squared error (SSE) (8), average absolute relative deviation (AARD) (9), and coefficient of determination (r^2^) (10). The RMSE values serve as indicators of the model’s efficiency by assessing the agreement between calculated values and experimentally measured values. On the other hand, MBE values are employed to ascertain the standard deviation between the predicted and measured values [36,37,38]. These statistical parameters were calculated using equations [39]. In addition, Yoon’s method of global sensitivity (8) was used to evaluate the direct influence of the input parameters on the output variables, which correspond to the weighting coefficients (w) within the ANN model [40,41]:

(4) $$\chi^{2}=\frac{\sum_{i=1}^{N}{\left(x_{exp,i}-x_{pre,i}\right)}^{2}}{N-n}$$

(5) $$RMSE={\left[\frac{1}{N}\cdot \sum_{i=1}^{N}{\left(x_{exp,i}-x_{pre,i}\right)}^{2}\right]}^{1/2}$$

(6) $$MBE=\frac{1}{N}\cdot \sum_{i=1}^{N}\left(x_{exp,i}-x_{pre,i}\right)$$

(7) $$MPE=\frac{100}{N}\cdot \sum_{i=1}^{N}\left(\frac{\left|x_{exp,i}-x_{pre,i}\right|}{x_{pre,i}}\right)$$

(8) $$SSE=\sum_{i=1}^{N}{\left(x_{exp,i}-x_{pre,i}\right)}^{2}$$

(9) $$AARD=\frac{100}{N}\cdot \sum_{i=1}^{N}\left|\frac{x_{exp,i}-x_{pre,i}}{x_{pre,i}}\right|$$

(10) $$r^{2}=1-\frac{\sum_{i=1}^{N}{\left(x_{exp,i}-x_{pre,i}\right)}^{2}}{\sum_{i=1}^{N}{\left(x_{exp,i}-\bar{x}\right)}^{2}},\bar{x}=\sum_{i=1}^{N}x_{exp,i}$$

where *N* represents the total number of data records, while *x_exp_*_,*i*_ and *x_pre,i_* are the experimental and model-predicted values, respectively.

## 3. Results and Discussion

### 3.1. SVM Modeling

The SVM models were created to predict the quality parameters of cookies with the addition of dehydrated peach based on the input parameters, including the percentage of dehydrated peach addition as well as the treatment for obtaining dehydrated peach (lyophilization and lyophilization with osmotic pretreatment). The models were created as regression type 1 with a training constant of 9. The epsilon measure of the model was set to 0.1, while the radial basis function (gamma value) was set to 0.50. The total number of model iterations was 10,000.

**Figure 3:**
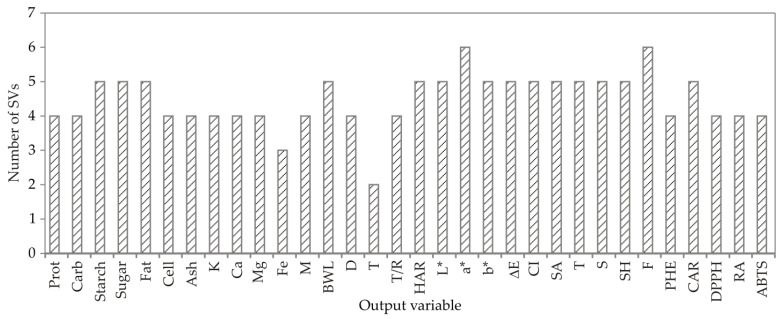
The number of SVMs.

On Figure 3 the number of support vector machines used to create SVM models for observed output variables is shown.

### 3.2. ANN Modeling

For all 32 of the observed responses, ANN models were conducted separately. The results of mathematical modeling for logical representation and better illustration were organized into six groups: the first group included the basic chemical compositions of the cookie samples (contents of protein, carbohydrate, starch, sugar, fat, cellulose, and ash); the second covered selected mineral contents (K, Ca, Mg, and Fe); the third group represented moisture content, baking weight loss, diameter, thickness, T/R ratio, and hardness; the fourth group included the color characteristics of the cookie samples (L*, a*, b*, and ΔE); the fifth group covered the sensorial properties of the cookie samples (color intensity, surface appearance, taste, smell, sensory hardness, and, fracturability); and finally the sixth group represented the antioxidant properties of the observed samples (total polyphenol content, total carotenoid content, antioxidative activity by DPPH, antioxidative activity by ABTS, and RP-reduction potential) [21].

The developed models for the first group of results, presented in Table 1, showcased favorable generalization properties, enabling accurate predictions of observed parameters. These predictions were based on the percentage of dehydrated peach addition and selection of the peach drying method (lyophilization and lyophilization with osmotic pretreatment). According to the calculations of the ANN models, the optimal configuration for the number of neurons in the hidden layers was determined to be 9, 4, 7, 7, 7,6, and 3, corresponding to the MLP 4-9-1, MLP 4-4-1, MLP 4-7-1, MLP 4-7-1, MLP 4-7-1, MLP 4-6-1, and MLP 4-3-1 network structures. Additionally, these models demonstrated a high coefficient of determination (r^2^), with values reaching 0.997, 0.992, 0.998, 0.999, 0.999, 0.997, and 0.999 during the training and testing phases, underscoring the robustness and validity of the model.

The neural network models for the second group of results, as optimally designed and presented in Table 2, demonstrated favorable generalization properties, facilitating the prediction of the observed parameters. Based on the calculations of the ANN models, the optimal configuration for the number of neurons in the hidden layers was determined to be 6, 4, 3, and 7, corresponding to the MLP 4-6-1, MLP 4-4-1, MLP 4-3-1, and MLP 4-7-1 network structures. Furthermore, these models exhibited high coefficients of determination with values of 0.999, 0.997, and 0.993 during the training and testing cycle, confirming the model’s validity.

The developed models for the third group of results, as presented in Table 3, indicated favorable generalization properties, facilitating accurate predictions of output responses. In compliance with the developed ANN models, the optimal configuration for the number of neurons in the hidden layers was 9, 7, 6, 8, 8, and 7, corresponding to the MLP 4-9-1, MLP 4-7-1, MLP 4-6-1, MLP 4-8-1, MLP 4-8-1, and MLP 4-7-1 network structures. Moreover, the developed ANN models demonstrated high coefficients of determination with values of 0.997, 0.992, 0.998, 0.999, 0.999, 0.997, and 0.999 during the training and testing phases, underscoring the robustness and validity of the model.

The models developed for the color properties of the observed cookie samples are detailed in Table 4. According to the calculations of the Artificial Neural Network (ANN) models, the optimal configuration for the number of neurons in the hidden layers was determined to be 6, 7, 6, and 6. These values correspond to the MLP 4-6-1, MLP 4-7-1, MLP 4-6-1, and MLP 4-6-1 network structures, respectively. Furthermore, these models exhibited high coefficients of determination, with values reaching 0.980, 0.962, 0.999, and 0.997 during the training and testing phases. This underscores the robustness and validity of the models in predicting and capturing the color properties of the dehydrated peach-enhanced cookie samples.

The models developed for the fifth group of results, as presented in Table 5, exhibited favorable generalization properties, enabling accurate predictions of the observed parameters. According to the calculations of the Artificial Neural Network (ANN) models, the optimal configuration for the number of neurons in the hidden layers was determined to be 3, 5, 3, 4, 8, and 6. These values correspond to the MLP 4-3-1, MLP 4-5-1, MLP 4-3-1, MLP 4-4-1, MLP 4-8-1, and MLP 4-6-1 network structures, respectively. Additionally, these models demonstrated high coefficients of determination, with values reaching 0.973, 0.990, 0.927, 0.936, 0.967, and 0.828, confirming the validity and reliability of the models in predicting the parameters for the fifth group of results.

The developed models for the sixth group of results are presented in Table 6. According to the calculations of the Artificial Neural Network (ANN) models, the optimal configuration for the number of neurons in the hidden layers was determined to be 4, 8, 7, 8, and 5. These values correspond to the MLP 4-4-1, MLP 4-8-1, MLP 4-7-1, MLP 4-8-1, and MLP 4-5-1 network structures, respectively. Moreover, the models demonstrated high coefficients of determination, with values reaching 0.996, 0.994, 0.995, 0.953, and 0.967, confirming the validity and reliability of the models in predicting the parameters for the fifth group of results.

### 3.3. The Accuracy of the Models

To numerically assess the accuracy of the presented SVM and ANN models, various performance metrics such as reduced chi-square (χ^2^), root mean square error (RMSE), mean bias error (MBE), mean percentage error (MPE), total squared error (SSE), average absolute relative deviation (AARD), and coefficient of determination (r^2^) were calculated, as shown in Table 7 for the SVM models and in Table 8 for the ANN models. 

According to the presented results, the SVM and ANN models successfully underwent minor lack-of-fit tests, indicating their effective prediction of the values for the analyzed parameters. Slightly higher coefficients of determination were obtained using SVM for the content of cellulose, Fe content, thickness, hardness, L*, a*, ΔE, color intensity, smell, sensory hardness, and fracturability. However, ANN models more efficiently predicated the majority of the observed parameters.

### 3.4. Global Sensitivity Analysis—Yoon’s Interpretation Method

**Figure 4:**
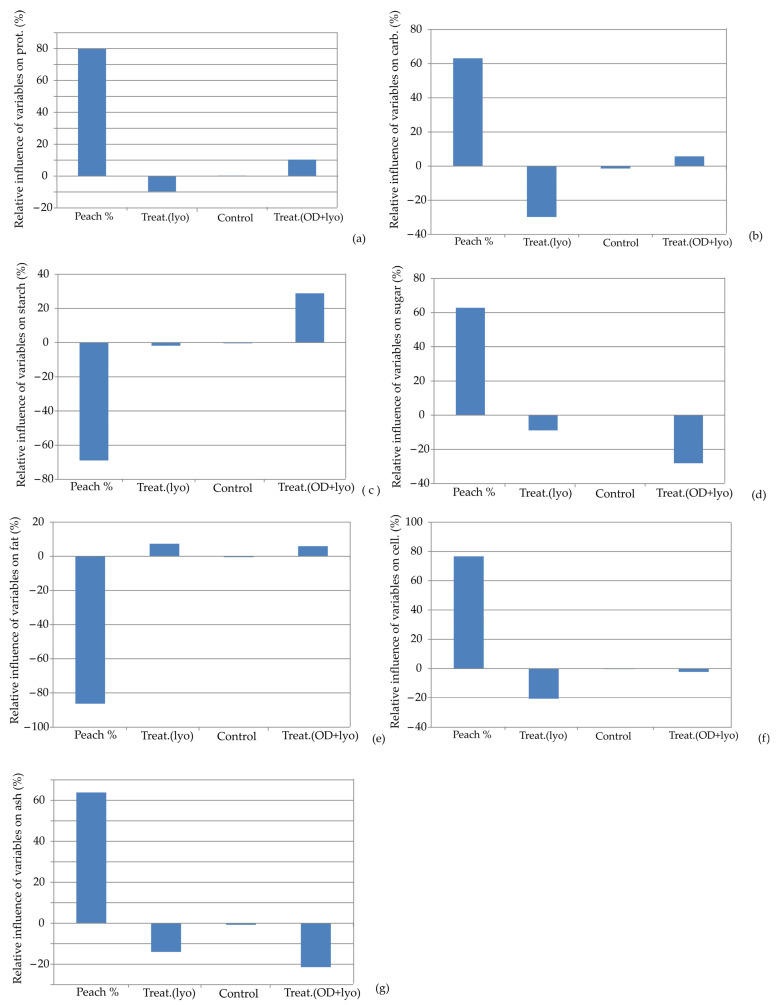
Relative importance of peach addition and treatment selection on: (**a**) protein content, (**b**) carbohydrate content, (**c**) starch content, (**d**) sugar content, (**e**) fat content, (**f**) cellulose content, and (**g**) ash content.

The influence of the input variables on the relative importance of the protein content, carbohydrates content, starch content, sugar content, fat content, cellulose content, and ash content for the ANN models is illustrated in Figure 4. According to Figure 4, the addition of dehydrated peach was the most influential parameter of all the observed parameters. It positively influenced protein content (+80.00%), carbohydrate content (+62.23%), sugar content (+61.89%), cellulose content (+78.99%), and ash content (+64.44%). On the other hand, it had a negative influence on starch content (−68.87%) and fat content (−86.49%).

**Figure 5:**
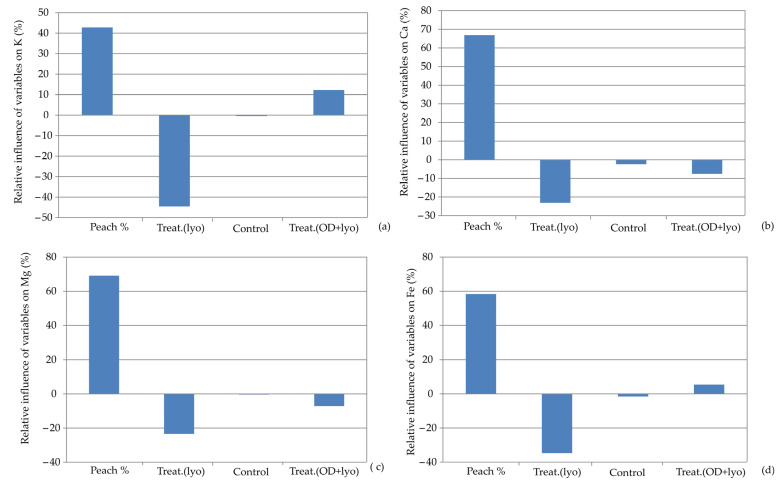
Relative importance of peach addition and treatment selection on: (**a**) K content, (**b**) Ca content, (**c**) Mg content, and (**d**) Fe content.

Figure 5 illustrates the impacts of the input variables (the percentage of dehydrated peach addition and the drying treatment employed in the preparation of the dehydrated peach) on the relative importance of K content, Ca content, Mg content, and Fe content in the developed Artificial Neural Network (ANN) models. It is noticed that the percentage of dehydrated peach addition was also the most influential parameter, inducing positive responses for K (+42.87%), Ca (67.86%), Mg (+68.35%), and Fe (+59.86%). Osmotic pretreatment had the most positive influence on the K content (+11.54%). The research by Lončar et al. [42] underlined that the addition of osmotically pre-treated and then lyophilized apples to muffin samples was the most influential factor affecting mineral composition, especially K content. Sugar beet molasses, with their high dry matter (80%) and specific nutrient content, are an excellent medium for osmotic pre-treatment, given their complex chemical composition that includes over 200 different inorganic and organic compounds [43].

**Figure 6:**
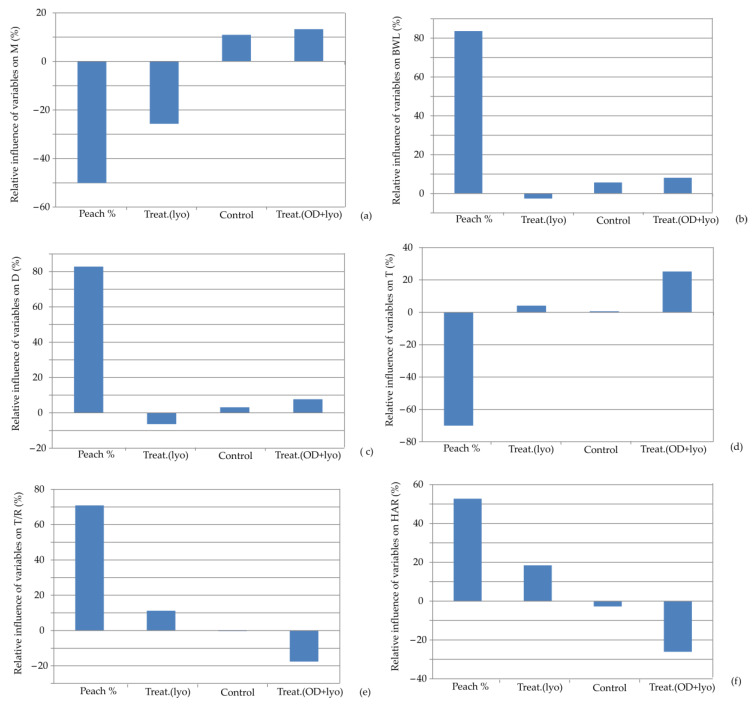
Relative importance of peach addition and treatment selection on: (**a**) moisture content, (**b**) baking weight loss, (**c**) diameter, (**d**) thickness, (**e**) T/R ratio, and (**f**) hardness.

The impact of input variables on the relative importance of moisture content, baking weight loss, diameter, thickness, T/R ratio, and hardness for the developed ANN models is presented in Figure 6. It is observed that the percentage of dehydrated peach addition is the most influential parameter. It affected negatively moisture content (−66.87%) and thickness (−47.56%), while it had the opposite effect on baking weight loss, diameter, T/R ratio, and hardness, with relative importance of +83.64%, +82.20%, +70.12%, and +53.13%, respectively. The research by Grigelmo-Miguel et al. [44] reported that the addition of peach dietary fiber in reduced-fat muffins increased their hardness and chewiness. However, springiness and cohesiveness were not different.

**Figure 7:**
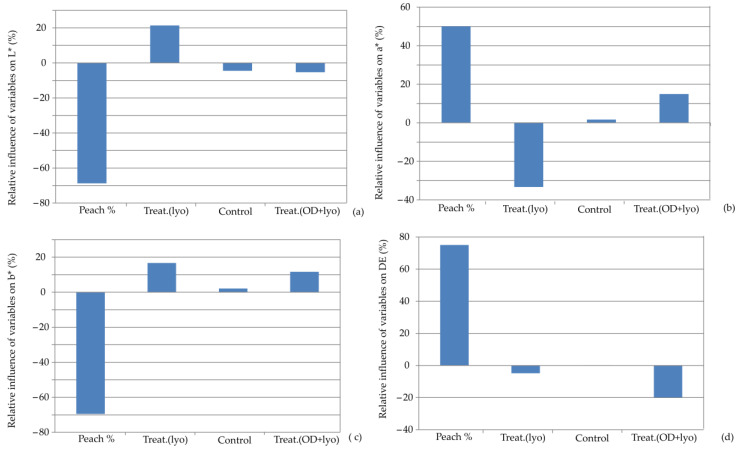
Relative importance of peach addition and treatment selection on: (**a**) L*, (**b**) a*, (**c**) b*, and (**d**) ΔE.

The influence of input variables on the relative importance of the color characteristics of the observed cookie samples is given Figure 7. The percentage of dehydrated peach addition was the most influential parameter, with negative influences on L* (−69.92%) and b* (−69.98%) while positively influencing a* (+50.00%) and ΔE (+77.00%). Drying treatments had the opposite effect on L*and a*, and these findings are in accordance with the results of Filipović et al. [21]. Salehi and Aghajanzadeh [45] highlighted that muffins incorporating varying amounts of peach dietary fiber (2%–10%) exhibited increased levels of moisture, protein, and minerals, along with fewer calories compared with the control sample. Nevertheless, the inclusion of peach dietary fiber led to a darker color in the muffins and an increase in both hardness and chewiness. The substitution of flour by dehydrated peach in the muffins increased their density and reduced the number of air pockets, thereby increasing the force needed for compression. These findings align with the research conducted by Grigelmo-Miguel [46], which demonstrated that incorporating various fruit powders into the batter formulation resulted in a firmer texture or increased hardness of the final products. Additionally, they are consistent with the findings of Mihaylova. [47], who observed that the texture quality parameters of wheat cookies decreased with higher levels of peach powder added to the mixture.

**Figure 8:**
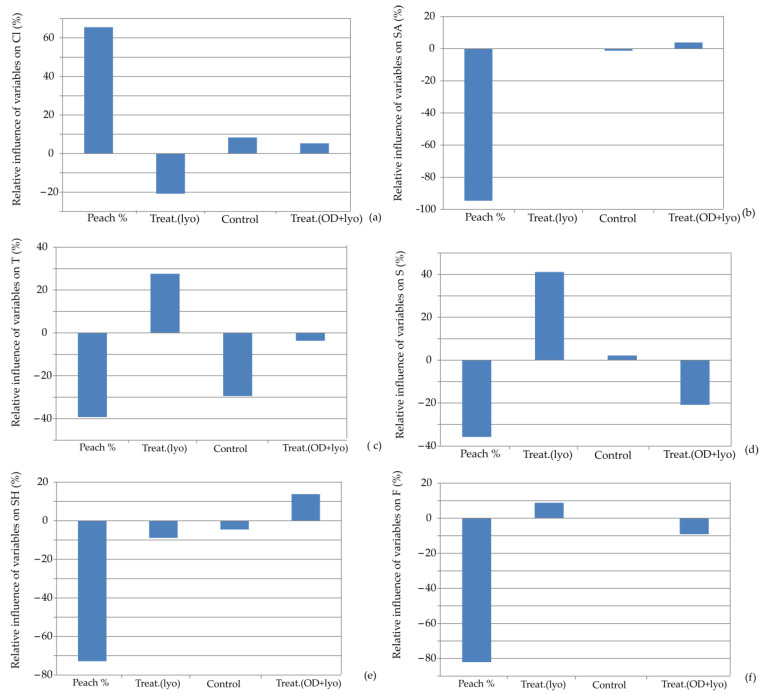
Relative importance of peach addition and treatment selection on: (**a**) color intensity, (**b**) surface appearance, (**c**) taste, (**d**) smell, (**e**) sensory hardness, and (**f**) fracturability.

The influence of input variables on the relative importance of the color intensity, surface appearance, taste, smell, sensory hardness, and fracturability for the developed ANN models is illustrated in Figure 8. It can be noted that the percentage of dehydrated peach addition is again the most influential parameter affecting the observed responses. It positively affected only color intensity while having negative impacts on the other responses. The significant change in the color of the cookies when the peach was dehydrated using the osmotic pre-treatment method can be attributed to the influence of molasses on the overall appearance of the cookie color [48]. Molasses, recognized for their dark hue, impart color to the dehydrated peach through solid gain. Additionally, molasses catalyze the development of Maillard reactions and caramelization, which further contributes to the overall change in cookie color [49].

**Figure 9:**
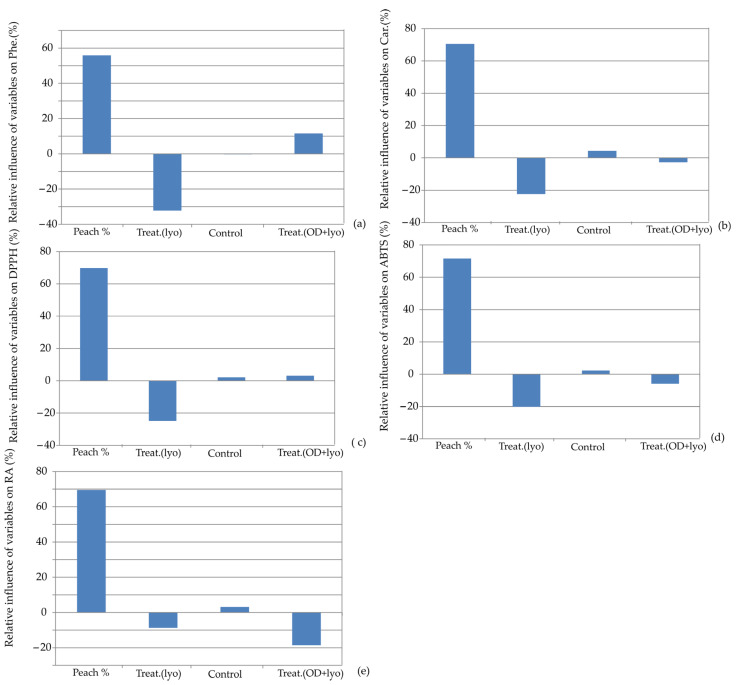
Relative importance of peach addition and treatment selection on: (**a**) total polyphenol content, (**b**) total carotenoid content, (**c**) antioxidative activity by DPPH, (**d**) antioxidative activity by ABTS, and (**e**) RP-reduction potential.

Furthermore, Figure 9 illustrates the relative importance of the total polyphenol content, total carotenoid content, antioxidative activity by DPPH, antioxidative activity by ABTS, and RP-reduction potential for the developed ANN models. Moreover, numerous researchers observed that fortifying cookies with polyphenol antioxidants sourced from various fruits and vegetables enhances their antioxidant properties [50,51].

### 3.5. Multi-Objective Optimization

The established Artificial Neural Network (ANN) models, encompassing all 32 examined parameters related to nutritive and technological quality responses were employed for Multi-Objective Optimization (MOO). The objective was to identify the optimal overall quality of the dehydrated peach-supplemented cookie samples. The MOO solution resulted in a Pareto front, indicating an improvement in one objective function without adversely affecting others [52]. Utilizing the genetic algorithm (GA), the MOO problem was tackled using a stochastic approach inspired by natural evolution incorporating mutation, selection, inheritance, and crossover [53]. The MOO computation was executed using the gamultiobj function in Matlab software version 12.5.0. The initial population was randomly generated and subjected to a set of points within the design space. The populations of subsequent generations were determined through a distance measure and the non-dominated ranking of individual points in the current generation [54,55].

The numerical tasks were handled separately for each constructed Artificia Neural Network (ANN) model through the application of Multi-Objective Optimization (MOO) calculations in Matlab. The MOO procedure was specifically crafted to pinpoint the optimal combinations of process parameters. Its goal was to maximize the output variables associated with both nutritive and technological quality within the ANN models. Constraints employed in the optimization process were applied within the experimental range of parameters, following the recommendations of Filipović et al. [21]. The optimization process for ANN models involved 400–500 generations, with a fixed population size of 1000 for each input variable across all models. The Pareto front comprised 12 to 20 points for the calculated ANN models. Ultimately, the optimum addition of OL peach to the cookie formulation was determined to be 15%. The resulting optimal sample is in accordance with the results obtained by Filipović et al. [21].

## 4. Conclusions

The employed Support Vector Machine and Artificial Neural Network models accurately predicted the quality parameters of cookies enriched with dehydrated peach, considering input factors such as the percentage of dehydrated peach and the treatment method (lyophilization and lyophilization with osmotic pretreatment in sugar beet molasses). The SVM models exhibited slightly higher coefficients of determination (r^2^ up to 0.981) and lower root mean square errors (RMSE down to 0.027) for specific parameters, while the ANN models demonstrated overall greater efficiency in predicting the majority of the 20 observed parameters (r^2^ reaching 1.000 and RMSE of 0.009). Sensitivity analysis underscored the significance of the percentage of dehydrated peach, positively impacting various aspects including protein, carbohydrate, sugar, cellulose, and ash contents while negatively affecting starch and fat contents. Osmotic pretreatment notably influenced potassium content. Baking characteristics, color properties, and sensorial attributes were primarily influenced by the presence of dehydrated peach. Multi-objective optimization using the genetic algorithm determined the optimal addition of dehydrated peach to be 15%, aligning with prior research. Future studies could focus on refining machine learning models, exploring hybrid approaches, and validating findings across diverse conditions to enhance the accuracy and applicability of models for optimizing cookie quality using dehydrated peach.

## Figures and Tables

**Table 1:** Artificial Neural Network model summary (performance and errors) for training, testing, and validation cycles for the contents of protein, carbohydrate, starch, sugar, fat, cellulose, and ash.

Output Variable	Net. Name	Performance		Error		Training Algorithm	Error Function	Activation	
		Train.	Test.	Train.	Test.			Hidden	Output
Protein	MLP 4-9-1	0.997	1.000	0.0007	0.00009	BFGS 5	SOS	Identity	Tanh
Carbohydrate	MLP 4-4-1	0.992	1.000	0.0004	0.009	BFGS 13	SOS	Tanh	Exponential
Starch	MLP 4-7-1	0.998	1.000	0.0021	0.0226	BFGS 54	SOS	Exponential	Tanh
Sugar	MLP 4-7-1	0.999	1.000	0.0031	0.0527	BFGS 32	SOS	Tanh	Logistic
Fat	MLP 4-7-1	0.999	1.000	0.0002	0.0001	BFGS 51	SOS	Exponential	Exponential
Cellulose	MLP 4-6-1	0.997	1.000	0.0005	0.011	BFGS 33	SOS	Logistic	Exponential
Ash	MLP 4-3-1	0.999	1.000	0.00002	0.0001	BFGS 24	SOS	Logistic	Tanh

Train.—training cycle, Test.—testing cycle.

**Table 2:** Artificial Neural Network model summary (performance and errors) for training, testing, and validation cycles for the contents of K, Ca, Mg, and Fe.

Output Variable	Net. Name	Performance		Error		Training Algorithm	Error Function	Activation	
		Train.	Test.	Train.	Test.			Hidden	Output
K	MLP 4-6-1	0.999	1.000	0.1000	0.0007	BFGS 32	SOS	Tanh	Exponential
Ca	MLP 4-4-1	0.997	1.000	0.0573	0.0000	BFGS 9	SOS	Logistic	Identity
Mg	MLP 4-3-1	0.991	1.000	0.292	0.101	BFGS 11	SOS	Logistic	Tanh
Fe	MLP 4-7-1	0.993	1.000	0.00004	0.0001	BFGS 15	SOS	Logistic	Logistic

Train.—training cycle, Test.—testing cycle.

**Table 3:** Artificial Neural Network model summary (performance and errors) for training, testing, and validation cycles for moisture, BWL, R, H, RH, and HAR.

Output Variable	Net. Name	Performance		Error		Training Algorithm	Error Function	Activation	
		Train.	Test.	Train.	Test.			Hidden	Output
M	MLP 4-9-1	0.962	1.000	0.243	2.543	BFGS 5	SOS	Identity	Identity
BWL	MLP 4-7-1	0.995	1.000	0.0298	0.0820	BFGS 17	SOS	Exponential	Tanh
D	MLP 4-6-1	0.977	1.000	0.091	0.016	BFGS 28	SOS	Exponential	Exponential
T	MLP 4-8-1	0.992	1.000	0.018	0.881	BFGS 16	SOS	Tanh	Tanh
T/R	MLP 4-8-1	0.996	1.000	0.007	0.112	BFGS 21	SOS	Logistic	Logistic
HAR	MLP 4-7-1	0.967	1.000	3.282.	2.701	BFGS 10	SOS	Identity	Identity

Train.—training cycle, Test.—testing cycle.

**Table 4:** Artificial Neural Network model summary (performance and errors) for training, testing, and validation cycles for L*, a*, b*, and ΔE.

Output Variable	Net. Name	Performance		Error		Training Algorithm	Error Function	Activation	
		Train.	Test.	Train.	Test.			Hidden	Output
L*	MLP 4-6-1	0.980	1.000	0.1533	3.676	BFGS 5	SOS	Exponential	Exponential
a*	MLP 4-7-1	0.962	1.000	0.0778	1.3278	BFGS 6	SOS	Identity	Logistic
b*	MLP 4-6-1	0.999	1.000	0.0084	0.571	BFGS 35	SOS	Logistic	Logistic
ΔE	MLP 4-6-1	0.997	1.000	0.169	2.240	BFGS 23	SOS	Tanh	Logistic

Train.—training cycle, Test.—testing cycle, Valid.—validation cycle.

**Table 5:** Artificial Neural Network model summary (performance and errors) for training, testing, and validation cycles for color intensity, surface appearance, taste, smell, sensory hardness, and fracturability.

Output Variable	Net. Name	Performance		Error		Training Algorithm	Error Function	Activation	
		Train.	Test.	Train.	Test.			Hidden	Output
CI	MLP 4-3-1	0.973	1.000	0.0231	0.1886	BFGS 10	SOS	Identity	Identity
SA	MLP 4-5-1	0.990	1.000	0.0289	0.0262	BFGS 14	SOS	Exponential	Tanh
T	MLP 4-3-1	0.927	1.000	0.756	0.134	BFGS 1	SOS	Identity	Tanh
S	MLP 4-4-1	0.936	1.000	0.072	0.0349	BFGS 4	SOS	Identity	Logistic
SH	MLP 4-8-1	0.967	1.000	0.118	0.0400	BFGS 3	SOS	Exponential	Identity
B	MLP 4-6-1	0.828	1.000	0.219	0.0333	BFGS 3	SOS	Exponential	Identity

Train.—training cycle, Test.—testing cycle.

**Table 6:** Artificial Neural Network model summary (performance and errors) for training, testing, and validation cycles for total polyphenol content, total carotenoid content, antioxidative activity by DPPH, antioxidative activity by ABTS, and RP-reduction potential.

Output Variable	Net. Name	Performance		Error		Training Algorithm	Error Function	Activation	
		Train.	Test.	Train.	Test.			Hidden	Output
PHE	MLP 4-4-1	0.996	1.000	0.0001	0.0000	BFGS 5	SOS	Logistic	Exponential
CAR	MLP 4-8-1	0.994	1.000	0.9142	7.7654	BFGS 13	SOS	Exponential	Identity
DPPH	MLP 4-7-1	0.995	1.000	0.004	0.0089	BFGS 10	SOS	Exponential	Identity
ABTS	MLP 4-8-1	0.953	1.000	9.090	4.446	BFGS 5	SOS	Identity	Tanh
RP	MLP 4-5-1	0.967	1.000	3.084	4.854	BFGS 36	SOS	Tanh	Identity

Train.—training cycle, Test.—testing cycle.

**Table 7:** Tests for the developed SVM model’s “goodness of fit”.

		χ^2^	RMSE	MBE	MPE	SSE	AARD	r^2^
SVM	Prot	0.001	0.027	0.007	0.324	0.006	0.163	0.968
	Carb	0.001	0.035	0.001	0.041	0.011	0.259	0.981
	Starch	0.122	0.330	−0.128	0.657	0.831	1.783	0.956
	Sugar	0.110	0.313	0.112	0.493	0.770	1.757	0.973
	Fat	0.007	0.081	−0.026	0.509	0.053	0.470	0.979
	Cell	0.013	0.108	0.040	0.852	0.091	0.649	0.981
	Ash	0.002	0.044	0.016	4.563	0.015	0.248	0.958
	K	103.246	9.580	3.222	3.004	732.514	50.264	0.969
	Ca	2.158	1.385	0.561	3.112	14.432	7.143	0.951
	Mg	2.125	1.374	0.522	2.441	14.551	7.363	0.967
	Fe	0.001	0.035	0.017	1.693	0.008	0.215	0.895
	M	0.801	0.844	−0.433	16.067	4.720	6.259	0.950
	BWL	0.670	0.772	0.280	5.296	4.659	6.142	0.966
	D	2.921	1.611	0.696	1.185	19.004	8.179	0.610
	T	1.774	1.256	−0.264	9.509	13.560	8.669	0.452
	T/R	1.027	0.955	0.040	11.038	8.198	7.064	0.327
	HAR	8.246	2.707	0.717	10.559	61.342	21.775	0.972
	L*	8.554	2.757	−0.994	5.070	59.531	15.842	0.927
	a*	0.610	0.736	0.362	3.954	3.703	4.571	0.909
	b*	1.937	1.312	−0.529	5.414	12.976	7.042	0.888
	ΔE	10.786	3.096	1.155	8.773	74.285	17.660	0.923
	CI	0.192	0.413	0.194	4.495	1.195	2.390	0.891
	SA	0.396	0.593	−0.223	16.690	2.718	4.703	0.961
	T	0.536	0.690	−0.353	20.248	3.167	4.785	0.876
	S	0.022	0.139	−0.028	3.347	0.167	1.053	0.981
	SH	0.098	0.295	−0.136	7.770	0.616	1.774	0.914
	B	0.247	0.469	−0.086	10.795	1.911	3.121	0.867
	PHE	0.013	0.106	0.045	19.904	0.083	0.434	0.744
	CAR	37.421	5.767	2.233	13.086	254.473	29.361	0.902
	DPPH	0.053	0.218	0.018	66.574	0.424	1.643	0.865
	RA	916.533	28.543	3.624	41.219	7214.037	201.384	0.830
	ABTS	822.893	27.046	10.830	30.537	5527.627	117.238	0.825

**Table 8:** Tests for the developed ANN model’s “goodness of fit”.

		χ^2^	RMSE	MBE	MPE	SSE	AARD	r^2^
ANN	Prot	0.001	0.033	0.030	0.541	0.002	0.269	0.982
	Carb	0.005	0.068	−0.017	0.054	0.038	0.341	0.909
	Starch	0.015	0.115	0.034	0.238	0.110	0.679	0.989
	Sugar	0.032	0.168	−0.050	0.284	0.232	0.975	0.988
	Fat	0.000	0.018	0.001	0.142	0.003	0.131	0.997
	Cell	0.006	0.075	−0.025	0.523	0.045	0.364	0.973
	Ash	0.000	0.009	0.000	1.488	0.001	0.060	0.996
	K	0.177	0.396	0.117	0.179	1.291	2.664	1.000
	Ca	0.100	0.299	0.005	1.133	0.803	2.200	0.994
	Mg	0.562	0.707	0.155	1.914	4.281	5.071	0.980
	Fe	0.000	0.011	0.005	0.719	0.001	0.088	0.983
	M	1.689	1.225	0.498	12.668	11.276	7.363	0.870
	BWL	0.093	0.288	−0.056	2.316	0.718	2.025	0.995
	D	0.168	0.386	−0.091	0.462	1.267	3.106	0.951
	T	0.472	0.648	0.103	2.462	3.679	2.900	0.839
	T/R	0.069	0.248	−0.017	2.380	0.549	1.257	0.955
	HAR	106.101	9.711	−2.571	64.001	789.330	49.474	0.571
	L*	18.522	4.058	0.338	4.778	147.144	20.395	0.732
	a*	0.800	0.843	−0.141	5.163	6.219	4.635	0.768
	b*	0.218	0.441	−0.071	1.983	1.703	2.817	0.977
	ΔE	11.916	3.255	−1.184	3.377	82.712	13.700	0.897
	CI	0.135	0.346	−0.084	5.366	1.015	2.254	0.880
	SA	0.064	0.238	0.044	4.701	0.494	1.751	0.984
	T	1.372	1.104	0.108	30.445	10.873	8.272	0.729
	S	0.144	0.357	−0.142	10.489	0.967	2.979	0.865
	SH	0.041	0.190	0.030	4.623	0.318	1.335	0.902
	F	0.402	0.598	0.053	15.452	3.189	4.665	0.660
	PHE	0.000	0.013	−0.006	9.086	0.001	0.065	0.991
	CAR	5.483	2.208	−0.715	13.945	39.256	13.930	0.969
	DPPH	0.005	0.068	−0.030	26.712	0.034	0.420	0.960
	RA	28.326	5.018	−1.850	8.088	195.822	32.000	0.987
	ABTS	334.897	17.254	10.065	83.982	1767.459	114.503	0.896

## Data Availability

The original contributions presented in the study are included in the article, further inquiries can be directed to the corresponding author.
